# Low-Dose Pre-Operative Botulinum Toxin A Effectively Facilitates Complex Ventral Hernia Repair: A Case Report and Review of the Literature

**DOI:** 10.3390/medicina57010014

**Published:** 2020-12-28

**Authors:** Ali P. Mourad, Marie Shella De Robles, Robert D. Winn

**Affiliations:** Department of Surgery, The Wollongong Hospital, New South Wales 2500, Australia; shella.derobles@gmail.com (M.S.D.R.); winnro251@gmail.com (R.D.W.)

**Keywords:** botulinum toxins, clostridium botulinum, incisional hernia, herniorrhaphy

## Abstract

*Background:* Complex ventral hernias following laparotomy present a unique challenge in that repair is hindered by the lateral tension of the abdominal wall. A novel approach to overcome this is the “chemical component separation” technique. Here, botulinum toxin A (BTA) is instilled into the muscles of the abdominal wall. This induces flaccid paralysis and effectively reduces tension in the wall, allowing the muscles to be successfully joined in the midline during surgery. We describe a method where a large incisional hernia was repaired using this technique and review the variations in methodology. *Case report:* A woman in her mid-40s developed a ventral hernia in the setting of a previous laparotomy for a small bowel perforation. Computed tomography (CT) of the abdomen demonstrated an 85 (Width) × 95 mm (Length) ventral hernia containing loops of the bowel. Pre-operative botulinum toxin A administration was arranged at the local interventional radiology department. A total of 100 units of BTA were instilled at four sites into the muscular layers of the abdominal wall under CT-fluoroscopic guidance. She underwent an open incisional hernia repair 4 weeks later, where the contents were reduced and the abdominal wall layers were successfully joined in the midline. There was no clinical evidence of hernia recurrence at 3-months follow-up. *Conclusion:* Low-dose BTA effectively facilitates the surgical management of large ventral incisional hernias. There is, however, significant variation in the dosage, concentration and anatomical landmarks in which BTA is administered as described in the literature. Further studies are needed to assess and optimise these variables.

## 1. Introduction

Incisional hernia occurrence is an unfortunate complication associated with open abdominal surgery. The previous incision presents an area of inherent weakness that is susceptible to separation in the face of intra-abdominal pressures. For many patients, this can be physically and psychologically debilitating, which is why many opt for subsequent operative management [1]. The goals of an elective incisional hernia repair are to create a tension-free closure of the hernia defect, prevent early recurrence and mitigate the risk of abdominal compartment syndrome and its associated sequelae.

Complex incisional hernias encountered after laparotomy surgery present a unique challenge. The lateral traction of the abdominal wall invariably augments the size of the hernia defect which further complicates repair [2]. Several definitions exist based on the hernia’s linear defect span, transverse diameter or significant loss of domain [3,4]. The essence of all these definitions, however, is the same—a ventral incisional hernia where primary fascial closure is unlikely to be possible without adjunctive techniques. The component separation technique first described in the early 1990s was one of the more common employed [5]. With this strategy, the muscular layers of the anterior abdominal wall are separated intraoperatively, effectively reducing the tension in the wall, allowing the edges to be joined in the midline [6].

More recently, the “chemical component separation” technique using botulinum toxin A (BTA) has been popularised. BTA is a neurotoxin with a variety of applications in medicine, including detrusor overactivity, anal fissures, hyperhidrosis and cosmetic interventions [7]. It works at the neuromuscular junction by inhibiting the release of acetylcholine, thereby inducing flaccid paralysis. The peak onset of action is approximately two weeks with duration of action lasting up to six months [8]. By injecting BTA into the muscular layers of the abdominal wall, the muscle relaxing properties of the agent are exploited, allowing tension-free closure of the hernia defect several weeks later. This obviates the needs to separate the layers intraoperatively, thus preserving the myofascial architecture of the abdominal wall.

In this article, we describe a case where low-dose BTA was used to facilitate closure of a complex ventral hernia. Informed consent was obtained from the patient for publication of this case report. We also explore the methodological variation in this novel technique as described in the literature.

## 2. Case Presentation

**Figure 1:**
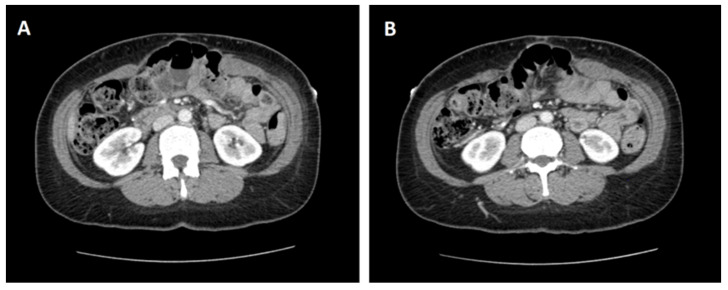
Transverse computed tomography (CT) slices at the level of L2 (**A**) and L3 (**B**) indicating a large ventral incisional hernia containing loops of the small and large bowel.

A woman in her mid-40s underwent an emergency laparotomy in the setting of an acute abdomen related to small bowel perforation. Her post-operative recovery was complicated by an admission to the intensive care unit for inotropic support and a surgical site infection requiring a return to theatres for washout. She was discharged approximately 4 weeks following her initial operation. In the coming months, she developed a swelling at the superior end of the laparotomy incision site. A computed tomography (CT) scan of the abdomen was obtained, demonstrating an 85 (Width) × 95 mm (Length) ventral incisional hernia containing loops of the small and large bowel without evidence of incarceration (Figure 1). Due to the size of the defect, a planned open incisional hernia repair facilitated by pre-operative BTA was arranged. Weight loss was also encouraged in the planning period to further increase the likelihood of operative success.

**Figure 2:**
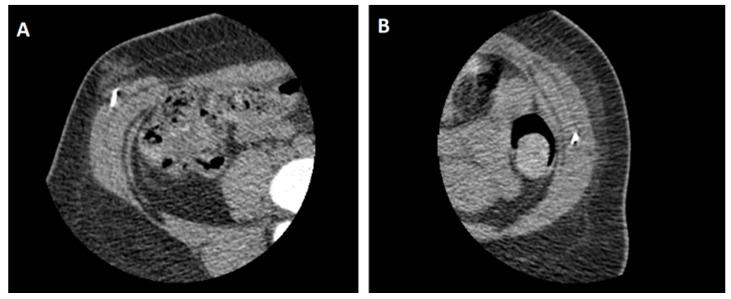
Still images of botulinum toxin A (BTA) instilled under CT-fluoroscopic guidance. The radiodense needle tip can be seen infiltrating into the right external oblique muscle (**A**) and the left internal oblique muscle (**B**).

The patient was booked into the local interventional radiology department 4 weeks prior to the scheduled operation date. Pre-procedural bloodwork was taken in the form of a full blood count and coagulation profile, excluding any coagulopathy which is a relative contraindication. On arrival, two injection sites were marked along the anterior axillary line on each side, equidistant between craniocaudal boundaries of the hernia (four sites in total). The patient was then positioned supine in a CT-scanner, the abdomen was prepared with 7.5% povidone-iodine and sterile drapes were applied around the surgical field. Strict asepsis was maintained throughout. Xylocaine 1% was infiltrated into the subcutaneous space at the marked injection sites. A total of 100 units of BOTOX^®^ (Allergan Pty Ltd, Gordon, NSW, Australia) were pre-diluted to 20 mL in 0.9% saline to a concentration of 5 U/mL. Using a 22-guage needle, 8–10 units of BTA were instilled by an interventional radiologist into the external oblique, internal oblique and transversus abdominus muscles under CT-fluoroscopic guidance at each injection site (Figure 2). The patient was monitored for an hour at bedside, had stable vital signs and was discharged with a plan to proceed with subsequent operative repair.

**Figure 3:**
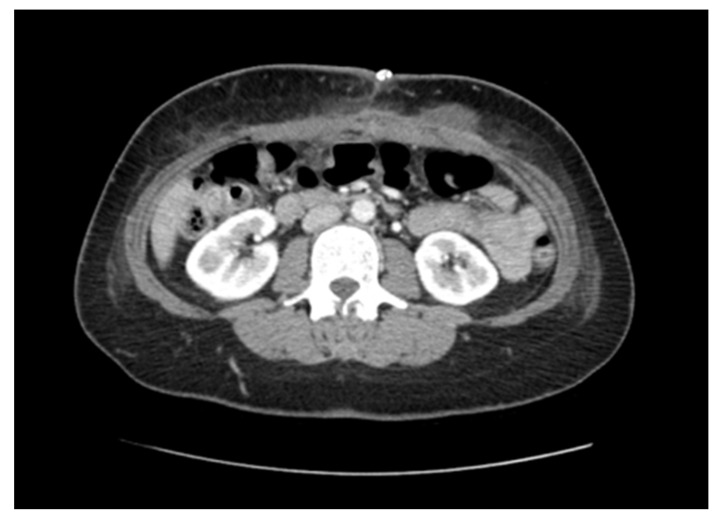
Axial CT imaging 2 weeks post-operatively indicating successful reduction in the hernia contents and repair of the abdominal wall defect. Post-surgical changes in keeping with the expected level at this time period are evident in the abdominal wall. A surgical clip is visible superficially overlying the wound.

An open hernia repair was performed 4 weeks later. The patient was positioned supine and a midline incision was made through the previous scar. The hernia sac was identified and dissected towards the edges to separate it from the anterior abdominal wall, followed by reduction into the abdominal cavity. Interrupted size 1 PDS sutures were used to successfully appose the fascial edges of the wall and close the hernia defect. Ultrapro Mesh (Ethicon Inc., Bridgewater, NJ, USA) was then fashioned and secured in the pre-fascial space with 2-0 PDS sutures to provide additional structural integrity. The umbilicus was then reconstructed and the skin closed with staples. An abdominal binder was left on for additional reinforcement and was subsequently removed along with the skin staples after 14 days. Post-operative CT scanning at 2 weeks demonstrated successful closure of the hernia defect and reduction in the abdominal contents (Figure 3). Follow-up at 3 months revealed no clinical evidence of hernia recurrence.

## 3. Discussion

In recent years, several innovative strategies have been developed in an attempt to offset the challenges that come with repairing complex ventral incisional hernias [9]. The “chemical component separation” technique was first described by Ibarra-Hurtado et al. in 2009 [10]. Since then, its efficacy has been validated through several prospective studies. Pre-operative BTA injection into the anterior abdominal wall muscles results in a reduction in the hernia defect diameter, a reduction in the abdominal wall thickness and an increase in the effective abdominal wall length, ultimately facilitating the closure of large ventral defects [11,12,13,14]. In this example, we were able to achieve primary fascial closure without complication and without evidence of recurrence at 3 months. Without BTA, a defect this size would have traditionally necessitated separating the abdominal wall layers [15,16].

The criteria for consideration of pre-operative BTA includes any ventral incisional hernia where primary fascial closure would be unlikely without adjunctive techniques. Hernia diameter appears to be the predominant determining factor given that this drives lateral tension in the abdominal wall [11]. In a study by Blair et al., a defect width above 8.3 cm was predictive of the requirement for component separation [16]. Cut-off diameters as low as 5 cm have been noted [13]. One other determinant is the presence of loss of domain, defined as the protrusion of a “significant” proportion of intra-abdominal contents beyond the natural contour of the abdominal wall. Although “significant” is frequently not defined, cut-offs of 15–20% have been described [12,14]. Absolute contraindications for the administration of pre-operative BTA include anaphylaxis to the product constituents and Myasthenia Gravis, while active coagulopathy is a relative contraindication [17].

In the literature, there is significant variability in the way the chemical component separation technique is implemented, first and foremost in the total dose of BTA administered. Factoring in differences in potency of the available BTA formulations [18], most studies administered a total cumulative dose equivalent to 200–300 U of BOTOX^®^ [11,12,13,19]. In the case described, a dose of only 100 U was used with success and further cases should explore the replicability of this outcome. We do, however, acknowledge that the mean transverse hernia diameter may have been larger in some of the aforementioned studies, ultimately influencing the decision to use larger dosages. Optimising the dose of BTA as a product of the size of the hernia defect could mitigate some of the adverse effects, which include a subjective weakness in coughing and sneezing [12,13]. This is an area yet to be explored.

Another disparity is the number and location of the BTA injection sites. We used a total of two sites at the anterior axillary line on each side, at points divided equally between the upper and lower boundaries of the hernia sac. Ibarra-Hurtado et al. described using five sites on each side, two over the mid-axillary line and three more anteriorly over the external oblique muscle [10]. Farooque et al. used three points on each side, marked equidistant along a vertical line from the inferior costal margin to the anterior super iliac spine [12]. The number and location of the sites required to maximise the degree of flaccid paralysis for any given dose is likely to also be dependent on the concentration of BTA instilled. Most articles have reported samples diluted to 2–5 U/mL, although up to 40 U/mL of equivalent BOTOX^®^ has been used [11,12,13,19]. BTA diluted in larger volumes distributes over greater areas within the muscular compartment [20].

The majority of studies used ultrasonography as the radiological tool to guide injections into the abdominal wall [11,12,13,19]. Ultrasonography has the advantages of being readily accessible and cost-friendly, having no radiation exposure and being relatively accurate in interventions of the musculoskeletal system [21]. In some cases, this was complemented by Electromyography (EMG) guidance to better direct BTA into the muscular layers [10,22]. We used real-time CT-fluoroscopy at the discretion of our institution’s interventionalist. In larger individuals, a thick subcutaneous layer may necessitate use of the convex probe during ultrasonography which compromises visibility of the instilling needle [21]. CT-fluoroscopy may be advantageous under these circumstances. Ultimately, the imaging modality used will depend on the availability of resources and the preference of the interventionalist.

One aspect that is relatively uniformly agreed upon is the latency between administering BTA and the time of surgery. Maximal paralysis is achieved after 2 weeks of administering BTA and begins to decline after 12 weeks [8]. Accordingly, most studies planned for surgery 2–4 weeks after administering the agent. One exception to this is a retrospective analysis performed by Zendejas et al., where the majority of patients were given BTA on the day of surgery. Despite administering BTA just prior to surgery, patients’ level of post-operative pain was less than that of controls [23]. This would suggest an additional analgesic benefit of BTA that is independent of its paralytic effect.

## 4. Conclusions

Through this case, we describe how pre-operative BTA effectively facilitates the repair of complex ventral hernias. Its ability to induce flaccid paralysis of the abdominal wall musculature can be exploited to create a tension-free closure, without separating the individual layers. At present, the technique is still in its infancy, and there is significant variability in how it is described in the literature. Priority should be given to randomised trials that look to optimise these variables.
